# Development and Psychometric Evaluation of the Critical Reflection Competency in Delirium Care Scale (CRCDCS) Among Iranian ICU Nurses

**DOI:** 10.1002/nop2.70818

**Published:** 2026-09-18

**Authors:** Fahimeh Magsoodi, Fatemeh Ghaffari

**Affiliations:** ^1^ Student Research Committee, Nursing Care Research Center, Health Research Institute Babol University of Medical Sciences Babol Iran; ^2^ Nursing Care Research Center, Health Research Institute Babol University of Medical Sciences Babol Iran; ^3^ Department of Nursing, Faculty of Medical Sciences Tarbiat Modares University Tehran Iran

**Keywords:** clinical competency, critical reflection, delirium, intensive care unit, nurses, reflective practice

## Abstract

**Aim:**

This study aimed to develop and psychometrically evaluate the Critical Reflection Competency in Delirium Care Scale (CRCDCS) among ICU nurses in Iran.

**Design:**

This exploratory mixed‐methods study was conducted in Iran between June 2025 and March 2026 based on the eight‐step scale development framework proposed by DeVellis and Thorpe.

**Methods:**

Item generation was performed using a systematic literature review and semi‐structured interviews with 15 ICU nurses. Following qualitative and quantitative assessment of content and face validity, the preliminary 19‐item CRCDCS was administered to 700 ICU nurses. Participants were randomly allocated into two equal subsamples for exploratory factor analysis (EFA; *n* = 350) and confirmatory factor analysis (CFA; *n* = 350). Psychometric evaluation included parallel analysis (PA), exploratory graph analysis (EGA), convergent and discriminant validity, random forest modeling (RFM), and reliability assessment using Cronbach's alpha (*α*), McDonald's omega (*ω*), intraclass correlation coefficient (ICC), and standard error of measurement (SEM).

**Results:**

EFA identified a three‐factor structure comprising Analytical–Evaluative Clinical Reflection (AECR), Person‐Centered Ethical Reflection (PCER), and Risk‐Oriented Preventive Reflection (ROPR), explaining 57.34% of the total variance. PA, EGA, and CFA supported the three‐factor model with excellent fit indices (*χ* ^2^/df = 1.14, CFI = 0.986, TLI = 0.984, RMSEA = 0.029, SRMR = 0.041). All factor loadings exceeded 0.40. Convergent and discriminant validity were confirmed. The scale demonstrated strong internal consistency (*α* = 0.876; *ω* = 0.861) and good test–retest reliability (ICC = 0.85, 95% CI: 0.82–0.91). RFM indicated that AECR items contributed most to the internal item‐set structure of the total score.

**Conclusion:**

The CRCDCS demonstrated sound internal structure, internal consistency, and test–retest reliability among ICU nurses in this Iranian sample. Further research is needed to establish external criterion validity, predictive validity, and measurement invariance before broader application of the scale.

**Impact:**

The CRCDCS is a multidimensional instrument for assessing ICU nurses' critical reflective competence in delirium care, capturing analytical, ethical, and preventive dimensions of reflective practice. Pending further validation, the scale may inform the design of educational programs and clinical training aimed at strengthening reflective decision‐making in delirium care within critical care settings.

**Reporting Method:**

This study was reported in accordance with the GRAMMS guidelines for mixed‐methods research and the COREQ checklist for the qualitative component.

**Patient or Public Contribution:**

No patient or public contribution.

**Abbreviations**
AVE: Average Variance Extracted
CFA: Confirmatory Factor Analysis
CFI: Comparative Fit Index
CMIN/DF: Chi‐square/degree‐of‐freedom ratio
CR: Construct Reliability
CRCDCS: Critical Reflection Competency in Delirium Care Scale
CVI: Content Validity Index
CVR: Content Validity Ratio
EGA: Exploratory Graph Analysis
GFI: Goodness of Fit Index
ICC: Intraclass Correlation Coefficient
IFI: Incremental Fit Index
MDC: Minimal Detectable Change
MG‐CFA: Multi‐Group Confirmatory Factor Analysis
MSV: Maximum Shared Variance
PA: Parallel Analysis
PCFI: Parsimonious Comparative Fit Index
PNFI: Parsimonious Normed Fit Index
RFM: Random Forest Model
RMSEA: Root Mean Square Error of Approximation
SEM: Standard Error of Measurement
SEM: Structural Equation Modeling
SFL: Standardised Factor Loading
*α*
: Cronbach's alpha coefficients
*Ω*
: McDonald's omega coefficient

## 1 Introduction and Background

Delirium is one of the most common and debilitating acute brain dysfunctions among critically ill patients and is recognised as a major challenge in intensive care unit (ICU) settings (Kotfis et al. 2018). It is associated with a wide range of adverse outcomes, including increased mortality, prolonged hospital stay, higher healthcare costs, and persistent cognitive impairment after discharge (Mart et al. 2021). These consequences highlight the urgent need to strengthen ICU nurses' competencies in the prevention, early detection, and effective management of delirium (Devlin et al. 2018; Hebeshy et al. 2024).

One of the core components of this competency is critical reflection, a reflective and analytical learning process that enables nurses to examine their clinical experiences in light of theoretical knowledge, research evidence, and professional ethical principles in order to improve decision‐making, enhance self‐awareness, and develop clinical judgement (Asselin 2011; Taylor 2000). In nursing, critical reflection is not only a mechanism for personal and professional growth, but also an essential process for improving the quality of patient care. Through reflective practice, nurses can bridge the gap between theoretical knowledge and clinical practice, integrate experiential and evidence‐based knowledge, and make more informed decisions in ethically and clinically complex situations (Patel and Metersky 2022; Subin and Sujin 2023).

Critical reflection competency among ICU nurses can be defined as the ability to systematically analyse clinical experiences, critically evaluate evidence‐based practices, identify and reconsider weaknesses in performance, and make ethical decisions in complex clinical contexts. By fostering analytical thinking, continuous self‐evaluation, and ethical reasoning, this competency may enhance professionalism, improve clinical decision‐making, and ultimately contribute to better outcomes for critically ill patients (Shin et al. 2022).

Despite the theoretical importance of critical reflection, existing instruments have important limitations for delirium care in ICU settings. The Critical Reflection Competency Scale developed by Shin et al. (2022) provides a framework for assessing critical reflection in clinical practice but was not designed specifically for delirium care (Shin et al. 2022). Similarly, the California Critical Thinking Disposition Inventory (CCTDI) (Facione et al. 2000), Critical Thinking Disposition Scale (Sosu 2013), Nursing Clinical Reasoning Scale (Liou et al. 2016). Reflective Thinking Questionnaire (Kember et al. 2000), and Reflective Thinking Scale for Healthcare Students and Providers (Liao and Wang 2019) assess broader aspects of critical thinking, clinical reasoning, or reflective thinking rather than delirium‐specific critical reflection competency.

Importantly, Chang et al. (2024) subsequently developed the Delirium Care Critical‐Thinking Scale for ICU nurses, addressing a closely related but conceptually distinct construct. Their five‐dimensional instrument focuses on knowledge application, information verification, logical reasoning, strategy selection, and open‐mindedness in delirium care. Although critical thinking and critical reflection overlap as higher‐order cognitive processes, critical reflection additionally involves examining one's own clinical experiences and practice, integrating evidence with professional experience, reconsidering ethical and person‐centered aspects of care, and reflecting on actions to inform subsequent practice (Chang et al. 2024). Thus, the present study addresses a more specific measurement gap: the lack of a delirium‐specific instrument explicitly designed to assess nurses' critical reflection competency, rather than the absence of delirium‐specific measures of related cognitive competencies.

This measurement gap underscores the need for a culturally grounded and context‐specific instrument with strong conceptual coherence and robust psychometric properties. Therefore, the present study aimed to develop and psychometrically evaluate the Critical Reflection Competency in Delirium Care Scale (CRCDCS) among ICU nurses in Iran.

## 2 Design and Methods

This exploratory mixed‐methods study was conducted between June 2025 and March 2026 across healthcare settings in Iran. The development of the CRCDCS was guided by the eight‐step scale development framework proposed by DeVellis and Thorpe (2021) (DeVellis and Thorpe 2021). The qualitative component of this study was reported in accordance with the Consolidated Criteria for Reporting Qualitative Research (COREQ) checklist (Tong et al. 2007).

### 2.1 Step 1: Define the Construct Domain

Critical reflection competency in nursing care is a multidimensional and integrative capability that encompasses cognitive–analytical, ethical, and interpretive dimensions, enabling nurses to systematically incorporate scientific evidence, professional experience, theoretical knowledge, and ethical values into clinical decision‐making through reflective practice and reflection‐in‐action (Dong et al. 2025; Lawrence 2011; Razieh et al. 2018; Shin et al. 2022). Unlike individual or education‐oriented reflection, this competency is directly manifested in complex and multidimensional clinical situations and contributes to improved patient outcomes by enhancing ethical sensitivity, reducing clinical errors, and strengthening situational decision‐making abilities (Bae and Roh 2024; Endacott et al. 2022; Teece et al. 2022; Yee 2023).

Within the context of delirium care in ICUs, this competency extends beyond routine clinical analysis. ICU nurses confronted with the dynamic, unpredictable, and often challenging nature of delirium are required to simultaneously interpret biological, psychological, social, and ethical dimensions of care while implementing strategies that preserve patient dignity, minimise restrictive interventions, and remain evidence‐based and clinically safe (Hamdin et al. 2025; Hebeshy et al. 2024; Vedsegaard et al. 2016).

Accordingly, in the context of the present study, critical reflection competency in delirium care was operationally defined as follows:

the ICU nurse's ability to systematically integrate scientific evidence, professional experience, ethical values, and theoretical knowledge through reflection‐on‐action and reflection‐in‐action with the aim of enhancing the accuracy of clinical decision‐making, improving quality of care, and ensuring the safety and dignity of patients with delirium in ICU settings.

### 2.2 Step 2: Generate an Item Pool

Item generation for the CRCDCS was conducted using both deductive and inductive approaches. Initially, a deductive approach based on a systematic review of the literature was employed to identify the theoretical dimensions of critical reflection competency in delirium care. Relevant studies were searched in PubMed, CINAHL, Scopus, Web of Science, ProQuest, and Google Scholar. Search strategies incorporated combinations of MeSH terms and keywords using the Boolean operators AND and OR, including ‘Critical Reflection Competency’, ‘Reflective Practice’, ‘Reflective Thinking’, ‘Delirium’, ‘ICU’, and ‘Scale Development’ or ‘Psychometric Validation’. Searches were conducted without time restrictions and included studies published in English and Persian.

Eligible studies were those examining critical reflection or reflective thinking within ICU settings or specifically in delirium care, as well as studies involving the development and psychometric evaluation of related scales among ICU nurses or comparable populations. Exclusion criteria included theoretical papers lacking clinical applicability, brief reports without sufficient methodological detail, and unpublished studies. Of the 130 records initially identified, 28 articles were retained for full‐text review after removal of duplicates and abstract screening. Ultimately, 21 studies met the inclusion criteria and were included in the final analysis. Content analysis of these studies resulted in the generation of 10 preliminary items.

To complement the findings derived from the systematic review and to ensure adequate representation of real‐world nursing behaviours and experiences related to critical reflection during delirium care, a qualitative study using conventional content analysis was conducted. Participants were selected from ICU nurses who had at least 1 year of ICU clinical experience, direct experience caring for at least five patients with delirium during the preceding 6 months, full‐time employment as professional nurses, and the ability to articulate and reflect upon clinical experiences. Participants also voluntarily agreed to participate in the study. All interviews were conducted by the corresponding author, Professor Ghaffari, a faculty member in nursing with extensive training and experience in qualitative data collection and content analysis. The interviewer had no clinical or hierarchical relationship with the participating nurses, and none of the participants were under the interviewer's supervision or evaluation in their clinical workplace, which minimised potential response bias.

Purposive sampling with maximum variation in terms of gender, work experience, and work shifts was employed, and 15 ICU nurses were recruited until data saturation was achieved. Data saturation was operationally defined as the point at which no new codes, themes, or meaning units emerged from two consecutive interviews. Sampling and data collection continued until this criterion was met, which occurred after the 15th interview. Data collection was conducted through individual semi‐structured interviews held via Skype in a quiet and private environment. Each interview lasted approximately 35–40 min and was audio‐recorded with participant consent.

Interview questions explored nurses' reflective experiences regarding delirium care, including reassessment of clinical decisions, recognition of discrepancies between evidence and practice, ethical evaluation of patient dignity and privacy, communication with families, identification of early warning signs, preventive reflection, and interpretation of longitudinal patient data and patterns.

Based on participants' responses, probing questions were used to deepen and clarify the information obtained. Following verbatim transcription of the interviews, data were analysed manually using the conventional content analysis approach proposed by Graneheim and Lundman (2004). Interview transcripts were read repeatedly to achieve immersion in the data. Meaning units relevant to the concept of critical reflection were identified and condensed into initial codes. Subsequently, codes were grouped into subcategories according to conceptual similarities and relationships. Through constant comparison and iterative review, subcategories were further integrated into overarching categories. This analytic process resulted in the generation of 20 items, 7 subcategories, and 3 principal categories: Analytical–Evaluative Clinical Reflection (AECR), Person‐Centered Ethical Reflection (PCER), and Risk‐Oriented Preventive Reflection (ROPR). All qualitative data collection, transcription, and analysis were conducted in Persian, the participants' native language, to preserve conceptual accuracy and nuance. The resulting items were subsequently translated into English using a forward–backward translation procedure: two bilingual experts independently translated the items forward into English, a third bilingual expert back‐translated them into Persian, and discrepancies were resolved through panel discussion to ensure semantic and conceptual equivalence. Table 1 provides an illustration of the item construction process at this stage.

**TABLE 1:** Example of item development for the Person‐Centered Ethical Reflection Category.

Category	Subcategory	Initial category	Codes	Final item	Participant quote
Person‐centered ethical reflection	Preservation of patient dignity and comfort	Ensuring personal respect and a calming environment	Properly covering the patient's body during procedures Limiting the presence of unnecessary individuals in the patient's room Creating a quiet and calm environment Documenting instances of compromised dignity in the medical record	During the review of care practices, I evaluate the extent to which respect, privacy, and physical comfort of patients with delirium were maintained based on observations and documented data, and I identify areas of deficiency	‘Whenever I perform a procedure for a patient with delirium, I make sure the patient's body is properly covered or use a bedside screen to provide privacy. I also try to limit unnecessary people in the room and maintain a calm environment. Whenever I noticed that these standards were not maintained, I documented the issue so that it could be corrected.’
	Monitoring the outcomes of therapeutic communication	Evaluating the impact of healthcare team interactions	Observing changes in family members' emotional responses Analysing the nurse's communication tone with the family Comparing family anxiety levels across different interactions Documenting factors contributing to changes in family reactions	Following each interaction with the family of a patient with delirium, I compare changes in their level of concern or anxiety based on observations or reports and identify the reasons in order to improve future communication strategies.	‘I remember a patient's family being extremely agitated. After I thoroughly explained the patient's condition and recovery process, they became calmer. However, when communication was rushed and lacked explanation, their anxiety increased. I noted these differences so I could improve my approach in future interactions.’
	Ethical–clinical evaluation of interventions	Analysing the appropriateness of intervention implementation	Assessing intervention‐related risks to patient safety Evaluating the suitability of interventions according to the patient's current condition Adhering to ethical standards during care implementation Recording suggested modifications in the patient record	After implementing an intervention, I reflect on its ethical and humanistic consequences in light of disease severity, level of consciousness, and care limitations, and document the findings to improve ethical approaches in similar future situations.	‘When I realised the patient was confused and exhausted, I performed the care procedure more gently and modified the approach slightly to make it safer and more humane. I also documented these modifications in the patient's record.’

#### 2.2.1 Trustworthiness

To ensure the rigour and trustworthiness of the qualitative findings, the criteria proposed by Guba and Lincoln (1994) were applied (Guba and Lincoln 1994). Credibility was established through purposive sampling with maximum variation, development of mutual trust through explanation of the study objectives and assurance of confidentiality prior to interviews, use of semi‐structured interviews with open‐ended and probing questions, and member checking was conducted by returning the extracted codes and categories to eight participants for review. Participants confirmed that the codes and categories accurately reflected their experiences; minor wording clarifications were made based on participant feedback, with no substantive changes to the category structure. Transferability was enhanced through rich and detailed descriptions of the research context, participant characteristics, and clinical settings, as well as presentation of sample codes, subcategories, and representative quotations from participants to demonstrate the applicability of findings to real‐world contexts.

Dependability was ensured by systematically documenting all stages of data collection, analytical decisions, and procedural modifications throughout the study, while portions of the coding and categorisation processes were independently reviewed by a second researcher. Confirmability was established through clear differentiation between raw data, initial codes, categories, and theoretical dimensions, enabling transparent tracing of the derivation of each item from raw data to higher‐level abstraction. In addition, all files, notes, and research documents were securely maintained to facilitate external auditing. Authenticity of the data was preserved by representing diverse nursing perspectives and experiences, including explicit descriptions of emotional, ethical, and humanistic concerns related to delirium care, and by presenting quotations that clearly reflected the reasoning underlying the extracted codes.

### 2.3 Step 3: Determine the Format for Measurement

The response format was determined concurrently with the item‐generation phase to ensure full alignment between item content and the response structure from the outset. Given that the construct of critical reflection competency in delirium care encompasses a continuum of analytical, ethical, and risk‐oriented behaviours, the selected response format needed to capture varying levels of behavioural frequency and intensity while allowing aggregation into a total competency score. Accordingly, a five‐point Likert scale ranging from never to always was adopted.

All items were formulated as behavioural and operational statements to assess the extent to which respondents actually performed reflective clinical behaviours rather than merely endorsing intentions or beliefs. The items were organised according to the theoretical dimensions of the construct, including Analytical–Evaluative Clinical Reflection (AECR), Person‐Centered Ethical Reflection (PCER), and Risk‐Oriented Preventive Reflection (ROPR), in order to facilitate a logical response flow, minimise respondent confusion, and reduce response fatigue and topic‐shifting bias during completion of the scale.

### 2.4 Step 4: Have Initial Item Pool Reviewed by Experts

At this stage, the operational definition of critical reflection competency in delirium care, together with its conceptual dimensions, was provided in written form to the expert panel to serve as the basis for item evaluation. Experts were selected using a purposeful expert selection strategy with consideration of disciplinary diversity. The panel consisted of 10 nursing faculty members with research experience in critical care nursing, clinical experience in ICUs, direct involvement in the management of patients with delirium, and prior experience in the development and psychometric evaluation of health‐related instruments.

Experts were asked to evaluate each item in terms of relevance to the construct, clarity and comprehensibility, and linguistic transparency. They were also encouraged to provide qualitative comments regarding wording modifications, semantic ambiguities, unintended interpretations, and potentially overlooked conceptual domains. Divergent interpretations among experts regarding item content were treated as warning indicators necessitating conceptual revision of the relevant items.

Quantitative content validity evaluation indicated that CVR values across the 30 initial items ranged from 0.40 to 1.00, with nine items falling below the acceptable threshold of 0.62 (Lawshe 1975) and subsequently removed. I‐CVI values for the retained items ranged from 0.80 to 1.00, exceeding the acceptable threshold of 0.79 (Polit and Beck 2006). The Scale‐level Content Validity Index using the averaging method (S‐CVI/Ave) was 0.93, and the Scale‐level Content Validity Index using the universal agreement method (S‐CVI/UA) was 0.86, both exceeding their respective acceptable thresholds of 0.90 and 0.80.

Following qualitative and quantitative content validity evaluation, all expert recommendations were integrated and reviewed by the research team with attention to conceptual clarity, cultural appropriateness, clinical applicability, content overlap, and theoretical coherence with the core construct. Decisions regarding item retention or deletion were not based solely on numerical indices; rather, the theoretical significance and conceptual contribution of each item to the construct dimensions were also considered. Consequently, only those items demonstrating unsatisfactory psychometric indices in addition to conceptual redundancy, excessive linguistic complexity, or limited applicability across clinical settings were removed. At this stage, nine items were deleted.

#### 2.4.1 Face Validity

Following expert review and item revision, the preliminary version of the CRCDCS was evaluated for face validity by 10 ICU nurses who met inclusion criteria similar to those of the target population but did not participate in the main psychometric study. Face validity was assessed qualitatively and quantitatively.

Participants evaluated each item regarding clarity, readability, simplicity, appropriateness of wording for the clinical setting, grammatical structure, and consistency with their professional experiences. Suggestions for revising ambiguous wording, unfamiliar terminology, or excessively lengthy statements were documented. Quantitative face validity was assessed using the *Impact Score* formula:

$$\text{Impact Score}=\text{Frequency}\;\left(\%\right)\times \text{Importance}$$

where Frequency referred to the percentage of participants rating an item as important or highly important, and Importance represented the mean importance score on a five‐point scale. Items with an impact score below 1.5 or judged irrelevant by participants were revised or removed (Polit and Beck 2006). At this stage, two items were removed: ‘After encountering challenging behaviors in a delirious patient, I reflect on whether the care provided was consistent with the patient's values, cultural beliefs, and personal identity’ and ‘Following disagreements between the family of a delirious patient and the healthcare team, I ethically re‐evaluate my role and determine whether my approach preserved the patient's interests and autonomy’. These items were primarily removed because participants demonstrated inconsistent interpretations of the abstract concepts embedded within them, the items showed partial conceptual overlap with other ethical‐reflection items, and their interpretation appeared difficult to standardise across nurses with diverse clinical backgrounds.

### 2.5 Step 5: Consider Inclusion of Validation Items

No additional items were incorporated into the preliminary version of the scale to assess response bias or external construct validation. At this stage, the development process focused exclusively on the core construct items, whereas assessment of additional forms of validity was deferred to subsequent psychometric evaluation phases.

### 2.6 Step 6: Administer Items to a Development Sample

Following final revision of the item pool, the preliminary version of the CRCDCS was administered to 700 ICU nurses employed in hospitals affiliated with Babol University of Medical Sciences and Mazandaran University of Medical Sciences, Iran. Sample size determination was based on the rule of thumb proposed by Nunnally and Bernstein, recommending a minimum of 10 participants per item (Nunnally and Bernstein 1994). This approach was adopted to minimise sampling error and ensure stability of inter‐item correlation patterns during psychometric analyses.

Inclusion criteria consisted of: (1) holding at least a bachelor's degree in nursing, (2) having a minimum of 1 year of clinical experience in a general ICU, and (3) being employed at one of the affiliated hospitals during the study period. The only exclusion criterion was failure to provide informed consent or incomplete submission of the study instruments.

Quota sampling proportional to the number of ICU nurses employed at each hospital was used to preserve representativeness of the target population. Initially, comprehensive lists of ICU nurses were obtained from the nursing management departments of both universities. Hospital‐specific quotas were then determined according to the relative distribution of nursing staff, and eligible participants were selected based on the inclusion criteria.

Data collection instruments included a demographic information form (age, gender, educational level, marital status, work experience, work shift, experience caring for patients with delirium, occupational position, and ICU experience) and the preliminary version of the CRCDCS. These instruments, together with the electronic informed consent form, were designed and distributed using the Porsline online survey platform. Survey links were disseminated through official communication channels managed by nursing supervisors via WhatsApp and Telegram. Participants completed the instruments after reviewing the study information and providing informed consent electronically.

For factor‐analytic procedures, the total sample was randomly divided into two equal subsamples: 350 participants for exploratory factor analysis (EFA) to identify the underlying factor structure of the scale, and 350 participants for confirmatory factor analysis (CFA) to evaluate model fit and confirm the proposed measurement structure.

### 2.7 Step 7: Evaluate the Items

#### 2.7.1 Exploratory Factor Analysis (EFA)

EFA was conducted using data from 350 ICU nurses. Prior to factor extraction, sampling adequacy was assessed using the Kaiser–Meyer–Olkin (KMO) index and Bartlett's Test of Sphericity. A KMO value of ≥ 0.80 was considered indicative of very good sampling adequacy, while a statistically significant Bartlett's test (*p* < 0.05) indicated suitability of the correlation matrix for factor analysis (Field 2024). Given the ordinal nature of Likert‐scale responses and the potential violation of normality assumptions, Principal Axis Factoring (PAF) was selected as the extraction method because of its robustness to non‐normal data distributions. The EFA was based on a Pearson correlation matrix. Pearson correlations were used because the five‐point Likert responses were treated as approximately continuous for the exploratory analysis. PAF was selected to provide robustness to potential departures from normality. PAF has been recommended as an appropriate approach for ordinal psychometric data and conditions in which multivariate normality cannot be fully assumed (Costello and Osborne 2005; Watkins 2018). Factor retention was determined primarily using parallel analysis, supplemented by the interpretability of the extracted factors, the magnitude of factor loadings, and the theoretical coherence of the resulting factor structure. A factor solution was retained when it was supported by parallel analysis and yielded theoretically interpretable dimensions with adequate item loadings.

The number of factors was determined using three criteria: eigenvalues > 1, scree plot inspection, and parallel analysis (Watkins 2018). Oblique Promax rotation was employed to account for potential correlations among latent factors (Fabrigar and Wegener 2012). Items with factor loadings below 0.40 were considered for conceptual review and potential modification (Hair et al. 2019). Factors with eigenvalues exceeding the 95th percentile of eigenvalues generated from parallel analysis were retained. Items with primary loadings below 0.40, or with cross‐loadings exceeding 0.32 on a secondary factor with a loading difference of less than 0.20 between the two factors, were flagged for conceptual review. Following this review, all initially flagged items were retained based on their theoretical relevance and absence of substantive conceptual overlap; no items were removed at this stage.

#### 2.7.2 Exploratory Graph Analysis (EGA)

EGA was performed to identify latent structural patterns and network‐based clustering among scale items. Initially, a partial correlation matrix was estimated using a Gaussian Graphical Model to identify direct relationships between variables (Epskamp et al. 2018). Network estimation was conducted using the graphical Least Absolute Shrinkage and Selection Operator (graphical LASSO) algorithm, which produces a sparse and stable network structure by eliminating weak associations through regularised estimation of the partial covariance matrix (Friedman et al. 2008). Because the CRCDCS items were measured using ordinal Likert‐type response categories, EGA was conducted using a polychoric correlation matrix to appropriately account for the ordinal nature of the data. EGA was used as a complementary, data‐driven approach to investigate the dimensional structure and community organisation of the items. Subsequently, the Walktrap algorithm was applied to detect clusters of items based on short random walks and local network density. To evaluate the stability and replicability of the network structure, Bootstrap EGA with repeated resampling was performed to assess the consistency of both the number of dimensions and item assignments across dimensions (Golino and Epskamp 2017).

#### 2.7.3 Confirmatory Factor Analysis (CFA)

CFA was conducted on an independent subsample of 350 ICU nurses distinct from the EFA sample to evaluate the fit of the hypothesised measurement model to the observed data. Given the ordinal nature of the scale items, the Weighted Least Squares Mean and Variance Adjusted (WLSMV) estimator was employed. This estimator is specifically recommended for ordinal and categorical data and does not require the assumption of multivariate normality (Flora and Curran 2004). Model fit was evaluated using the following indices: Chi‐square to degrees of freedom ratio (*χ* ^2^/df), Comparative Fit Index (CFI), Tucker–Lewis Index (TLI), Root Mean Square Error of Approximation (RMSEA), and Standardised Root Mean Square Residual (SRMR). Values of CFI and TLI > 0.90 together with RMSEA and SRMR < 0.08 were interpreted as indicative of acceptable model fit (Hair et al. 2019; Hu and Bentler 1999). Standardised factor loadings were examined for all items. Items with factor loadings below 0.50 or substantial cross‐loadings on theoretically unrelated factors were considered for conceptual reassessment.

#### 2.7.4 Scoring of Scale Items

The CRCDCS consists of 19 items distributed across three dimensions: Analytical–Evaluative Clinical Reflection (AECR), Person‐Centered Ethical Reflection (PCER), and Risk‐Oriented Preventive Reflection (ROPR). Responses to each item are scored using a five‐point Likert scale ranging from never to always (1 = never, 2 = rarely, 3 = sometimes, 4 = often, and 5 = always). For interpretive purposes, total scores were divided into three approximately equal‐width bands across the possible score range (19–95): low (19–44), moderate (45–70), and high (71–95) competency. These bands represent a conventional equal‐range categorisation intended to facilitate score interpretation and have not been empirically or clinically validated as diagnostic cut‐off points (Supporting Information Appendix A).

#### 2.7.5 Tests of Reliability

Three complementary approaches were used to evaluate the reliability of the CRCDCS. First, Cronbach's alpha coefficients were calculated for each dimension and for the total scale to assess internal consistency. Values of *α* ≥ 0.70 were considered acceptable, whereas values ≥ 0.80 indicated desirable reliability (Hair et al. 2009). Second, temporal stability was assessed using a test–retest design with 30 participants who completed the scale twice over a two‐week interval (Polit‐O'Hara and Yang 2016). Test–retest reliability was evaluated using the Intraclass Correlation Coefficient (ICC) based on a two‐way mixed‐effects model with absolute agreement. According to Koo and Li (2016), ICC values ≥ 0.75 indicate good reliability and values ≥ 0.90 indicate excellent reliability (Koo and Li 2016). Third, McDonald's omega (*Ω*) was calculated based on standardised factor loadings derived from the CFA model as a more robust alternative to Cronbach's alpha. Omega values ≥ 0.70 were considered acceptable and values ≥ 0.80 were considered desirable (Dunn et al. 2014).

#### 2.7.6 Convergent Validity (AVE‐Based) and Discriminant Validity

As no external criterion instrument was administered alongside the CRCDCS, convergent validity in this study refers specifically to model‐based convergent validity derived from the CFA measurement model, rather than criterion‐related convergent validity based on correlation with an external instrument. Convergent validity was supported by AVE values exceeding 0.50 for all three factors, indicating that each factor explained a substantial proportion of the variance in its associated items (Fornell and Larcker 1981), and by CR values greater than 0.70, demonstrating satisfactory composite reliability of the latent constructs (Hair et al. 2019). Discriminant validity was further supported by the Fornell–Larcker criterion. The square roots of AVE for AECR (0.746), PCER (0.732), and ROPR (0.737) exceeded their respective inter‐factor correlations, which were 0.364, 0.350, and 0.420. These findings indicate that, despite the substantive conceptual relationships among the three dimensions, each dimension retained sufficient empirical distinctiveness and was not redundant with the other dimensions. This differentiation is particularly relevant for multidimensional constructs associated with clinical judgement and professional decision‐making (Asselin and Fain 2013; Levett‐Jones et al. 2010). As no external criterion instrument was administered alongside the CRCDCS, the convergent validity evidence was model‐based (AVE‐derived) rather than criterion‐related; assessment against an independent, previously validated instrument remains an important direction for future research.

#### 2.7.7 Random Forest Modelling (RFM)

Random forest regression (Breiman 2001) was conducted as a supplementary exploratory analysis to examine the relative contribution of individual items and first‐order dimensions to the internal score structure of the CRCDCS. The analysis included the 19 items and, in a separate analysis, the three first‐order dimension scores, with the continuous total CRCDCS score used as the outcome. Relative importance was quantified using the percentage increase in mean squared error (%IncMSE) following permutation of each item. Because the predictors and outcome were derived from the same item set, the findings were interpreted only as descriptive evidence of the internal contribution structure of the scale and not as evidence of external predictive validity.

#### 2.7.8 Standard Error of Measurement (SEM)

The Standard Error of Measurement (SEM) was calculated to estimate the degree of random measurement error in observed test scores and to assess the precision with which observed scores reflect true scores (American Educational Research Association 2018). SEM is derived from the standard deviation of observed scores and the reliability coefficient of the scale; lower SEM values indicate greater measurement precision and score stability (Nunnally and Bernstein 1994). In psychometric evaluation, SEM provides additional evidence regarding the ability of an instrument to distinguish accurately between respondents and complements conventional reliability coefficients by offering a more comprehensive assessment of measurement quality. Systematic inclusion of SEM in psychometric analyses contributes meaningfully to evaluation of instrument precision and trustworthiness (Harvill 1991).

### 2.8 Step 8: Optimise Scale Length

To determine whether scale reduction was warranted, item–total correlations, Cronbach's alpha if item deleted, and communality indices were calculated for all items using reliability analysis procedures. Analyses were conducted in accordance with DeVellis' recommendations, and only items whose removal did not reduce reliability or improved psychometric performance were considered eligible for deletion. Results demonstrated that all items exhibited acceptable item–total correlations and adequate shared variance, and removal of any item failed to improve reliability indices, including Cronbach's alpha and McDonald's omega. Accordingly, the scale length was retained without modification, and all 19 items were preserved in the final version of the CRCDCS.

#### 2.8.1 Assessment of Normality and Outliers

Assessment of data normality and outliers was performed separately at both univariate and multivariate levels. Multivariate outliers were identified using Mahalanobis distance with a significance threshold of *p* < 0.001. In addition, multivariate kurtosis was assessed using Mardia's coefficient, with values greater than 20 considered indicative of violation of the multivariate normality assumption (Kline 2023).

### 2.9 Data Analysis

During EFA, factor extraction was conducted using Principal Axis Factoring (PAF) with Promax oblique rotation. These analyses were performed using SPSS version 26. For CFA, the Weighted Least Squares Mean and Variance Adjusted (WLSMV) estimator was implemented in R to account for the ordinal nature of the five‐point Likert‐scale items. In addition, EGA was conducted in the RStudio environment to estimate and visualise the network structure among scale items. As a supplementary exploratory analysis, the Random Forest algorithm was implemented using the randomForest package in R to examine the relative contribution of each item and dimension to the total competency score.

## 3 Results

### 3.1 Demographic Characteristics of Participants

The study sample consisted of 700 participants. Regarding sex distribution, 374 (53.4%) of the participants were male, and 326 (46.6%) were female. Regarding marital status, the majority of participants were married (*n* = 396, 56.6%), followed by single (*n* = 198, 28.3%), divorced (*n* = 66, 9.4%), and widowed participants (*n* = 40, 5.7%). With respect to educational attainment, most participants held a bachelor's degree in nursing (*n* = 613, 87.6%), while 87 (12.4%) held a master's degree. Regarding job position, 413 participants (59.0%) were clinical nurses, 262 (37.4%) were shift supervisors, and 25 (3.6%) were head nurses. Notably, the vast majority of participants (*n* = 669, 95.6%) reported prior experience caring for patients with delirium, whereas only 31 (4.4%) had no such experience.

### 3.2 Psychometric Properties

#### 3.2.1 Exploratory Factor Analysis

**FIGURE 1:**
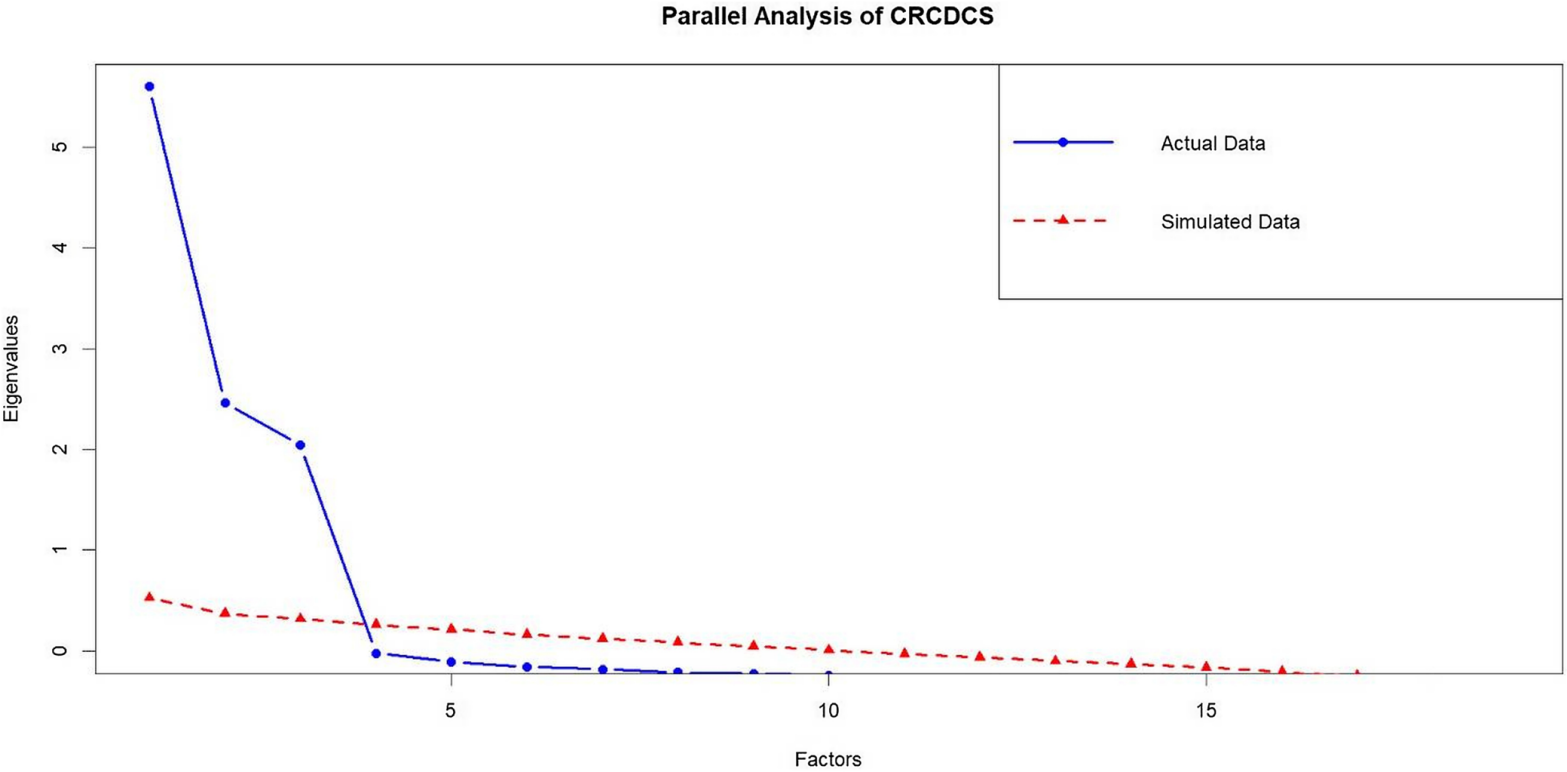
Parallel analysis for determining the optimal number of extractable factors.

Sampling adequacy analysis demonstrated a KMO value of 0.912, indicating excellent suitability of the data for factor analysis. Furthermore, Bartlett's test of sphericity was statistically significant (*χ* ^2^ = 3559.18, *p* < 0.001), suggesting the presence of sufficiently strong inter‐item correlations to support factor extraction. EFA identified a three‐factor structure for the CRCDCS, consisting of Analytical–Evaluative Clinical Reflection (AECR), Person‐Centered Ethical Reflection (PCER), and Risk‐Oriented Preventive Reflection (ROPR). These three latent factors accounted for 20.38%, 19.37%, and 17.59% of the total variance, respectively, collectively explaining 57.34% of the variance in critical reflection competency in delirium care. All 19 items demonstrated strong primary factor loadings, ranging from 0.633 to 0.853, with no substantial cross‐loadings observed across the three factors (Table 2). The complete pattern matrix, including both primary factor loadings and cross‐loadings, is provided in Table S1. In addition, the results of parallel analysis supported the three‐factor solution identified through EFA (Figure 1).

**TABLE 2:** Rotated factor pattern matrix (Promax rotation).

Factor	Item	Factor loading	Communality	Variance explained (%)	Eigenvalue
Analytical–evaluative clinical reflection	1. Following the assessment of patients with delirium based on specific protocols, I analyse the causes of deviations from these protocols within the healthcare team and document the findings.	0.683	0.457	20.38	3.87
	2. By reviewing delirium‐related care events, I separately analyse the factors influencing sustained cognitive improvement or deterioration, as well as acute clinical factors, and document the findings.	0.746	0.565		
	3. Through systematic evaluation of medication management, environmental conditions, and interprofessional interactions, I identify practices inconsistent with established evidence and document specific corrective recommendations to improve team performance.	0.719	0.525		
	4. By comparing the trajectory of delirium severity and disease progression with the patient's individual characteristics, I identify and document key factors requiring reconsideration of clinical interventions.	0.853	0.705		
	5. When screening results are inconsistent with the patient's clinical presentation, I analyse the possible causes of this discrepancy and document the findings to improve the accuracy of future assessments.	0.845	0.719		
	6. By analysing cognitive and functional documentation of patients with delirium after discharge, I identify areas requiring revision in care protocols and formally document corrective recommendations.	0.633	0.412		
	7. By comparing patients' cognitive, functional, and survival outcomes with initial predictions, I identify the causes of discrepancies and use the findings to refine future clinical decision‐making.	0.700	0.511		
Person‐centered ethical reflection	8. During the review of care practices, I evaluate the extent to which respect, privacy, and physical comfort of patients with delirium were maintained based on observations and documented data, and I identify areas of deficiency.	0.802	0.632	19.37	3.68
	9. Following each interaction with the family of a patient with delirium, I compare changes in their level of concern or anxiety based on observations or reports and identify the reasons in order to improve future communication strategies.	0.732	0.575		
	10. After implementing an intervention, I reflect on its ethical and humanistic consequences in light of disease severity, level of consciousness, and care limitations, and document the findings to improve ethical approaches in similar future situations.	0.733	0.562		
	11. I analyse patient records and family reports to identify sociocultural patterns influencing interaction and cooperation, and I apply the findings in designing communication and supportive interventions.	0.838	0.693		
	12. I analyse the influence of patients' or families' stated preferences and perspectives on care decisions and use the findings to improve the healthcare team's decision‐making process in similar future situations.	0.757	0.578		
	13. After implementing care interventions, I reflect on their impact on the comfort, dignity, and sense of safety of patients with delirium from the perspective of their lived experiences and family reports, and I use the findings to improve person‐centered care approaches.	0.830	0.668		
Risk‐oriented preventive reflection	14. Upon admission of high‐risk patients, I identify, prioritise, and document predisposing factors for delirium.	0.758	0.579	17.59	3.34
	15. I identify early behavioural or cognitive signs, such as transient attention deficits, and predict the onset of delirium by documenting and monitoring changes over time.	0.718	0.510		
	16. By reviewing the patient's care plan, I identify interventions that may contribute to the development or exacerbation of delirium and document the rationale for selecting or excluding each intervention.	0.826	0.654		
	17. By comparing documented data from similar patients, I identify predictive patterns of delirium and apply them in designing preventive interventions for future patients.	0.750	0.542		
	18. I analyse sequential physiological and cognitive changes in patients prior to the onset of delirium and document early warning indicators as future monitoring patterns.	0.685	0.511		
	19. I compare and analyse the results of repeated screenings in high‐risk patients to identify patterns of risk changes and, accordingly, revise and refine future monitoring and preventive intervention plans.	0.733	0.583		

#### 3.2.2 Confirmatory Factor Analysis

To evaluate model fit, the fit indices indicated excellent fit of the proposed model, with CMIN/DF = 1.14, RMSEA = 0.029, TLI = 0.984, CFI = 0.986, and SRMR = 0.041 (Table 3).

**TABLE 3:** Model fit indices for the confirmatory factor analysis of the CRCDCS.

CFA model	*χ* ^2^	Df	*p*	CMIN/df	RMSEA (CL90%)	CFI	TLI	SRMR
First‐order model	170.14	149	0.113	1.14	0.029 (0.002–0.044)	0.986	0.984	0.041
Second‐order model	170.14	149	0.113	1.14	0.029 (0.002–0.044)	0.986	0.984	0.041

**FIGURE 2:**
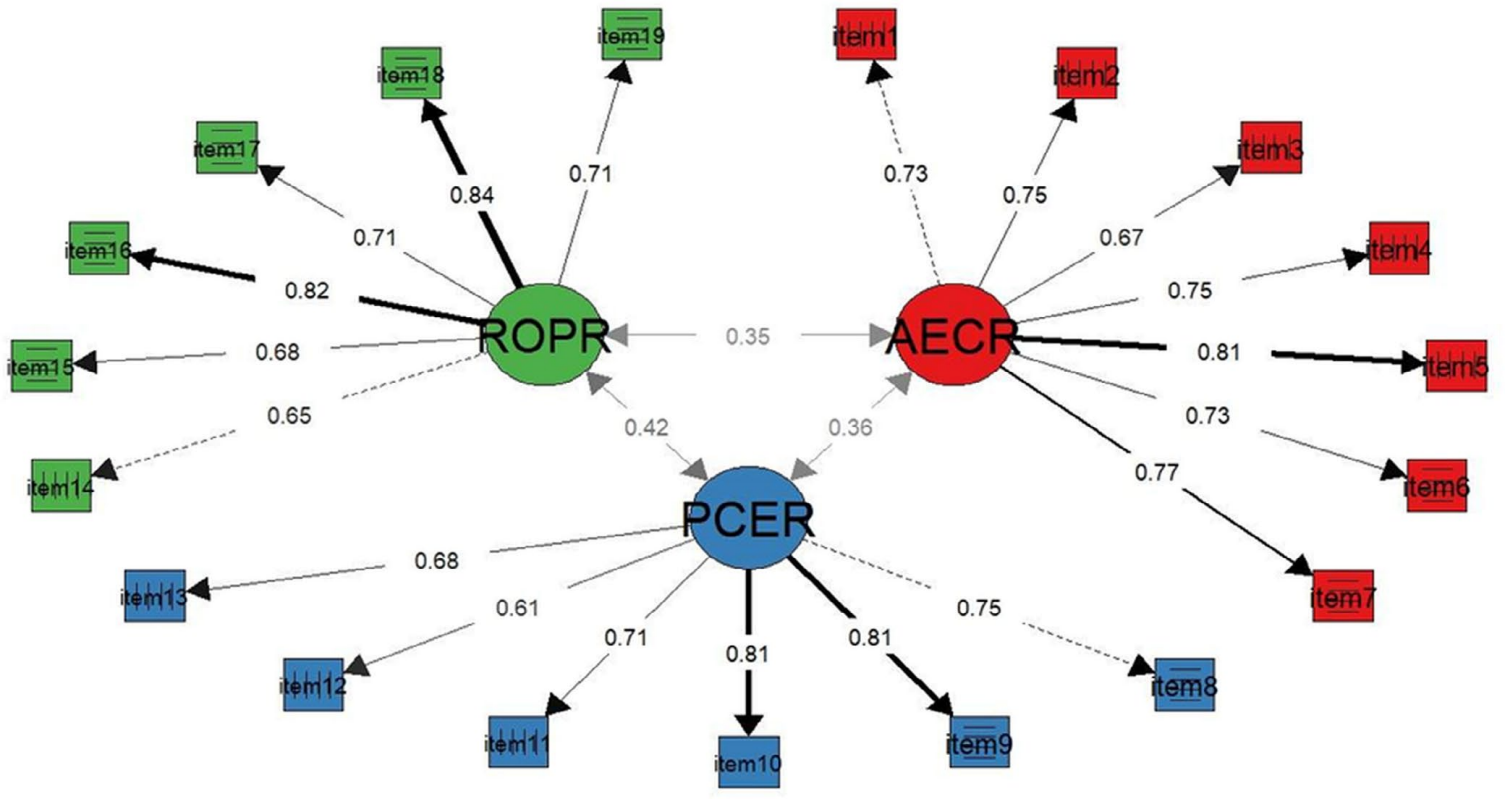
First‐order confirmatory factor analysis of the CRCDCS.

Figure 2 presents the standardised factor loadings between the items and latent factors of the CRCDCS within the first‐order confirmatory factor analysis model. All estimated factor loadings exceeded the recommended threshold of 0.40, indicating adequate relationships between the items and their corresponding latent constructs.

**FIGURE 3:**
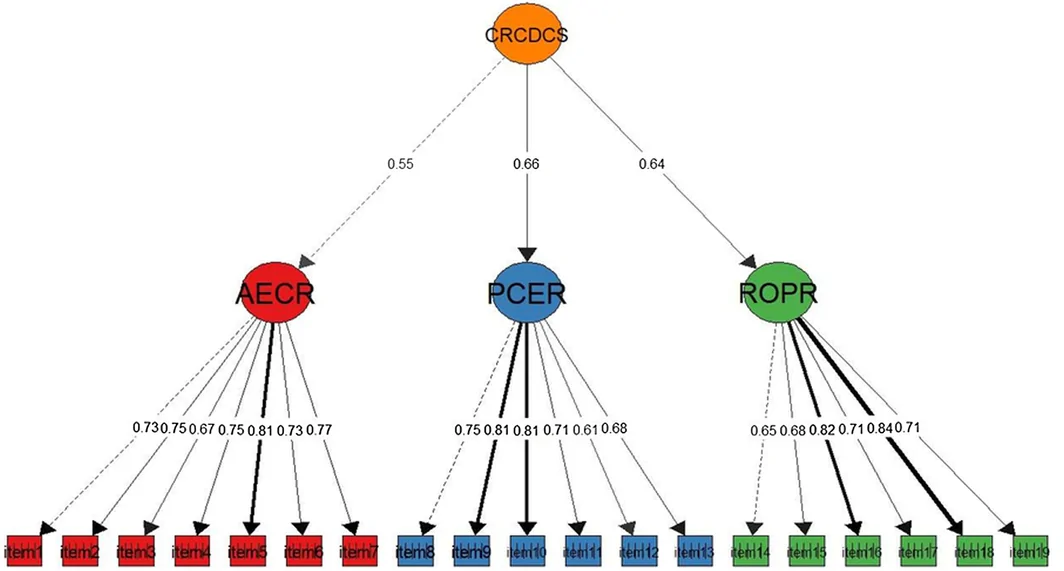
Second‐order confirmatory factor analysis of the CRCDCS.

A second‐order confirmatory factor analysis was conducted to examine the hypothesised higher‐order structure of the CRCDCS. In this model, Analytical–Evaluative Clinical Reflection (AECR), Person‐Centered Ethical Reflection (PCER), and Risk‐Oriented Preventive Reflection (ROPR) were specified as first‐order factors loading onto a single higher‐order factor representing overall Critical Reflection Competency in Delirium Care. All three first‐order factors showed positive and substantial standardised factor loadings on the higher‐order construct, as presented in Figure 3.

Because the second‐order model contained exactly three first‐order factors, it is statistically equivalent to the correlated three‐factor model. Consequently, the fit indices for the first‐order correlated model and the second‐order model were identical (CMIN/DF = 1.14, RMSEA = 0.029, TLI = 0.984, CFI = 0.986, and SRMR = 0.041). This equivalence is a mathematical property of a second‐order model with exactly three first‐order factors. Therefore, the identical fit indices should not be interpreted as evidence that the two models provide independently distinguishable empirical fits. Rather, the second‐order specification provides a hierarchical representation of the same underlying covariance structure, consistent with the hypothesised overarching construct of Critical Reflection Competency in Delirium Care. Accordingly, both the three dimension‐specific scores and the overall CRCDCS score were retained for subsequent interpretation.

### 3.3 Convergent Validity (AVE‐Based) and Discriminant Validity

The findings demonstrated that all Average Variance Extracted (AVE) values for the three latent factors exceeded the recommended threshold of 0.50, supporting satisfactory convergent validity. In addition, all Composite Reliability (CR) values were above 0.70, indicating adequate reliability of the latent constructs (Table 4). Discriminant validity was assessed using the Fornell–Larcker criterion by comparing the square root of the AVE for each factor with its correlations with the other factors. The square roots of AVE were 0.746 for AECR, 0.732 for PCER, and 0.737 for ROPR. The standardised correlations among the three latent factors were 0.364 between AECR and PCER, 0.350 between AECR and ROPR, and 0.420 between PCER and ROPR. In all cases, the square root of the AVE for each factor exceeded its correlations with the other factors, supporting discriminant validity among the three dimensions.

**TABLE 4:** Convergent validity, discriminant validity, internal consistency, and stability of the CRCDCS.

Factor	*α*	*Ω*	ICC (95% CI)	CR	AVE
AECR	0.876	0.878	0.76 (0.72–0.80)	0.898	0.556
PCER	0.845	0.850	0.80 (0.77–0.85)	0.873	0.536
ROPR	0.850	0.854	0.75 (0.71–0.82)	0.876	0.543
Total	0.876	0.861	0.85 (0.82–0.91)	—	—

### 3.4 Reliability

The Cronbach's alpha and McDonald's omega coefficients for the overall CRCDCS were 0.876 and 0.861, respectively, indicating satisfactory internal consistency. Among the latent dimensions, AECR demonstrated the highest reliability, with a Cronbach's alpha of 0.876 and an omega coefficient of 0.878. The consistency between alpha and omega coefficients across all factors further supported the reliability of the instrument. The intraclass correlation coefficient (ICC) for the total scale was 0.85 (95% CI: 0.82–0.91), reflecting satisfactory temporal stability (Table 4).

#### 3.4.1 Exploratory Graph Analysis

**FIGURE 4:**
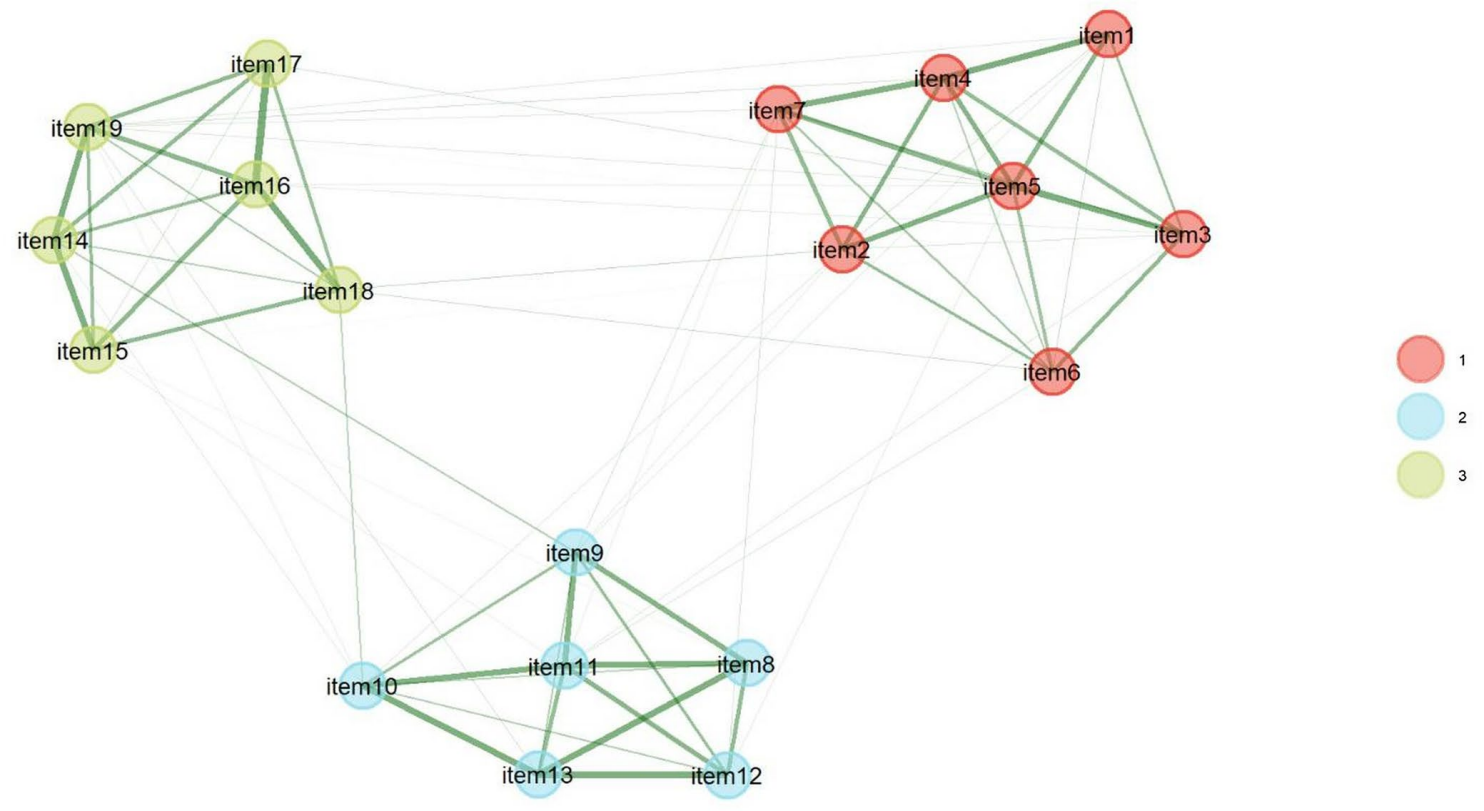
Network analysis results for the CRCDCS.

The EGA findings were consistent with the three‐factor structure identified through EFA. A total of 73 significant edges were identified within the estimated network, with a network density of 0.289, indicating a moderate degree of connectivity among the variables. The mean edge weight was 0.117, with weights ranging from −0.027 to 0.391, reflecting relationships varying from weak to moderately strong across the network. The Walktrap algorithm identified three distinct communities among the items. Notably, all items associated with each latent factor were completely clustered within their corresponding community, such that the distribution of items across the three communities (AECR, PCER, and ROPR) was fully consistent with the theoretical three‐factor model. This convergence provides strong evidence supporting the structural validity of the instrument. Examination of dimensionality further indicated that the data were not unidimensional, thereby supporting the multidimensional nature of the construct and the adequate distinction between its latent dimensions. The Total Entropy Fit Index (TEFI = −28.37) further confirmed the satisfactory fit of the network model. Overall, the network analysis findings were highly consistent with the factor analytic results and further strengthened the construct validity of the CRCDCS (Figure 4).

#### 3.4.2 Item and Dimension Contribution Analysis (Random Forest)

As a supplementary exploratory analysis, random forest regression showed that the CRCDCS items differed in their relative contribution to the total score. Because the predictors and outcome were derived from the same item set, these findings should not be interpreted as evidence of external predictive validity based on the %IncMSE index, the items ‘When screening results are inconsistent with the patient's clinical presentation, I analyze the possible causes of this discrepancy and document the findings to improve the accuracy of future assessments’, ‘I compare and analyze the results of repeated screenings in high‐risk patients to identify patterns of risk changes and, accordingly, revise and refine future monitoring and preventive intervention plans’, and ‘By comparing the trajectory of delirium severity and disease progression with the patient's individual characteristics, I identify and document key factors requiring reconsideration of clinical interventions’ showed the highest relative contribution to the total score. In contrast, the items ‘Following the assessment of patients with delirium based on specific protocols, I analyze the causes of deviations from these protocols within the healthcare team and document the findings’ and ‘By reviewing delirium‐related care events, I separately analyze the factors influencing sustained cognitive improvement or deterioration, as well as acute clinical factors, and document the findings’ showed the lowest relative contribution. The remaining items showed moderate levels of contribution.

**FIGURE 5:**
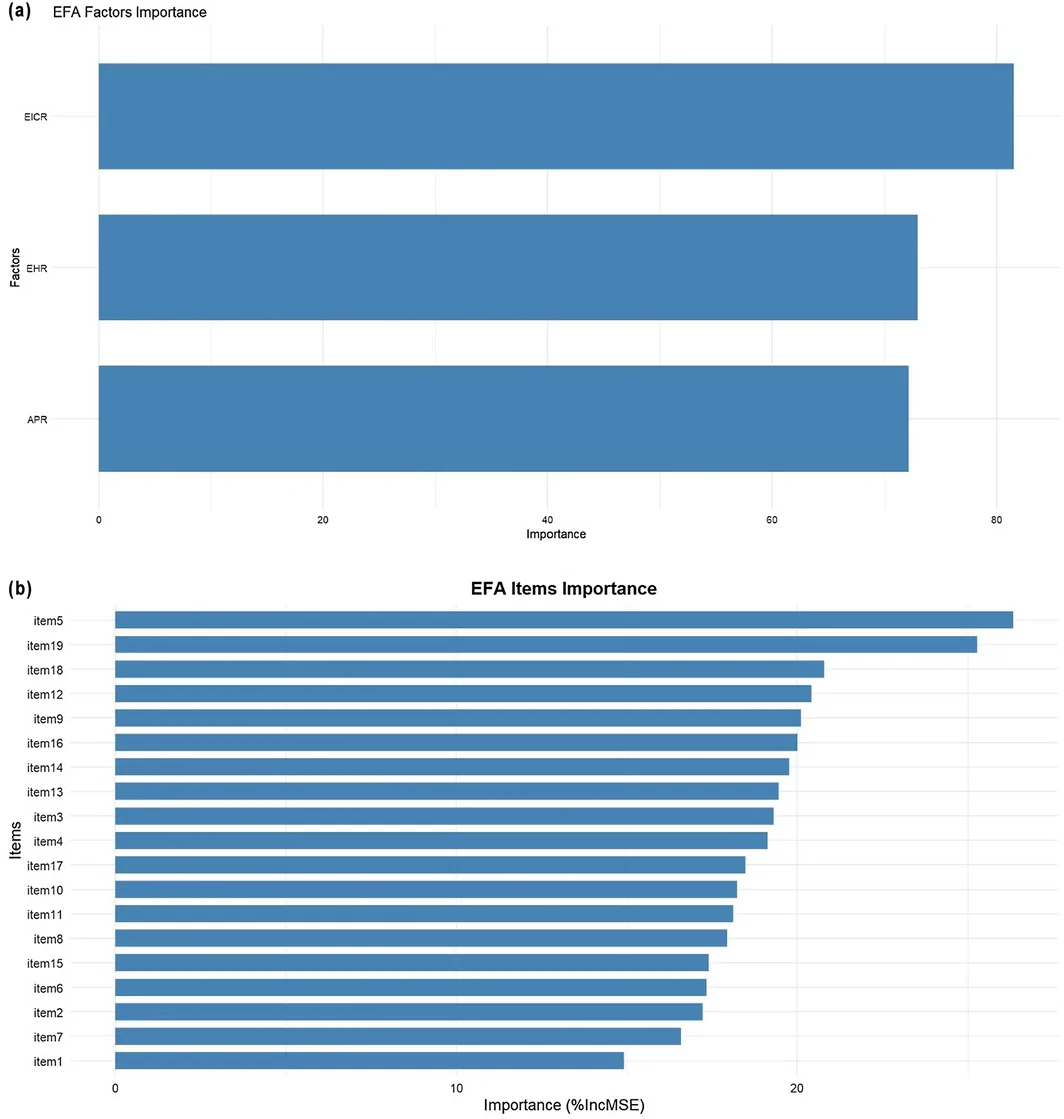
Random forest analysis results for examining the relative importance of CRCDCS items and dimensions in predicting the total score.

At the dimensional level, the three CRCDCS subscale scores showed different levels of relative contribution to the total CRCDCS score. AECR showed the largest relative contribution (%IncMSE = 80.27), followed by PCER (71.43) and ROPR (68.01). These findings indicate that all three dimensions contributed to the internal score structure of the CRCDCS, although AECR showed the highest relative contribution. Given the mathematical overlap between the predictors and the total score, these findings should be interpreted cautiously and should not be considered evidence of external predictive validity (Figure 5).

#### 3.4.3 Standard Error of Measurement (SEM)

The SEM values were 0.42 for AECR, 0.51 for PCER, 0.48 for ROPR, and 0.49 for the total CRCDCS score. These relatively low SEM values indicate a limited degree of random measurement error and suggest adequate precision of the observed scores in assessing critical reflective competence in delirium care.

## 4 Discussion

This study was conducted to develop and psychometrically evaluate the CRCDCS among nurses working in Iranian ICUs. The findings provide evidence regarding the internal structure, reliability, and selected aspects of construct validity of the instrument, while other forms of validity, particularly predictive and external criterion‐related validity, remain to be established.

The EFA findings demonstrated that the scale comprised a three‐factor structure consisting of AECR, PCER, and ROPR. The satisfactory KMO value and the significant Bartlett's test confirmed sampling adequacy and the suitability of the correlation matrix for factor analysis (Hair et al. 2009; Watkins 2021). The three extracted factors collectively explained 57.34% of the total variance of the construct, indicating satisfactory structural adequacy of the instrument in representing the concept of critical reflective competence (Hair et al. 2009). These findings suggest that critical reflective competence in delirium care extends beyond purely diagnostic or technical skills and also encompasses analytical, ethical, relational, and preventive dimensions of care, consistent with contemporary perspectives on delirium management that emphasise the integration of reflective thinking, clinical judgement, ethical sensitivity, and early risk recognition (Hayhurst et al. 2016; Pun et al. 2019; Wilson et al. 2020).

The AECR factor explained the largest proportion of the variance among the three extracted dimensions. This finding indicates that AECR accounted for a relatively greater share of the common variance captured by the factor solution in the present sample. Given the complex and fluctuating nature of delirium, the prominence of this dimension within the statistical structure may be compatible with the need for continuous monitoring, reassessment, interpretation of changing clinical information, and modification of care interventions (Devlin et al. 2018; Hayhurst et al. 2016). This interpretation is also broadly consistent with evidence emphasising the role of clinical reasoning and analytical reflection in delirium recognition and management (Pun et al. 2021; Rice et al. 2014). However, the greater proportion of explained variance should not be interpreted as evidence that AECR is inherently more important than PCER or ROPR from a theoretical or clinical perspective. Explained variance is a characteristic of the observed factor structure and does not establish hierarchical importance among dimensions. Accordingly, AECR is considered a prominent dimension within the empirical structure identified in this study rather than the definitive core or most important dimension of critical reflective competence. Establishing whether any dimension has greater theoretical or clinical importance will require further evidence from external criterion‐related validity, longitudinal studies, and investigations linking individual dimensions to relevant clinical or professional outcomes.

The PCER factor reflected the humanistic, ethical, and relationship‐centered dimensions of caring for patients with delirium, emphasising analysis of the patient's lived experience, family involvement, and the ethical consequences of care interventions, consistent with contemporary person‐centered care frameworks (Lee‐Steere et al. 2024; Lin et al. 2022; McCormack and McCance 2016; Santana et al. 2018). The ROPR factor emphasised identification of risk factors, early symptom recognition, and predictive thinking, aligning with the shift in the delirium literature from reactive treatment toward preventive models of care (Barnes‐Daly et al. 2017; Hshieh et al. 2015; Inouye et al. 2014; Marcantonio 2017). Taken together, the three‐factor structure suggests that critical reflective competence in delirium care is a dynamic, multidimensional construct integrating analytical, ethical, and preventive dimensions within a coherent conceptual framework (Benner 2001; Kuiper et al. 2009; Schön 2017; Tanner 2006; Timmins 2008).

Parallel analysis supported the extracted three‐factor structure and provided stronger evidence for factor retention than traditional eigenvalue‐based criteria (Howard and O'Sullivan 2025; Watkins 2021). The CFA findings demonstrated excellent model fit for the first‐order, correlated three‐factor model, with CFI and TLI values exceeding 0.95 and RMSEA and SRMR values below recommended thresholds (Hair et al. 2019; Kline 2023). A second‐order model, in which AECR, PCER, and ROPR were specified as first‐order factors loading onto a single higher‐order construct, was also examined. Because this model included exactly three first‐order factors, it is statistically equivalent to the correlated three‐factor model the two parameterisations describe the same covariance structure among the three dimensions and therefore necessarily yield identical fit indices. Accordingly, the present CFA evidence should not be interpreted as demonstrating the superiority of a hierarchical structure over a correlated three‐factor structure; rather, it confirms only that the three dimensions are meaningfully correlated. Distinguishing empirically between these two structures would require a fourth first‐order factor or an independent validation sample, and we recommend this as a direction for future research.

Given this statistical equivalence, the rationale for calculating and interpreting a total CRCDCS score is primarily theoretical rather than statistical. Consistent with Tanner's (2006) model of clinical judgement and Schön's (2017) conceptualisation of reflective practice, critical reflection is enacted in clinical settings as an integrated process in which analytical, ethical, and anticipatory reasoning operate simultaneously rather than as isolated, additive skills (Schön 2017; Tanner 2006). A total score is therefore intended to represent this integrated expression of critical reflective competence in delirium care, providing a practical, parsimonious index for screening, benchmarking, and monitoring change over time, while the three subscale scores remain available for more granular, dimension‐specific assessment and feedback.

Convergent validity was supported by AVE values exceeding 0.50 for all three factors, indicating that each factor explained a substantial proportion of the variance in its associated items (Fornell and Larcker 1981), and by CR values greater than 0.70, demonstrating satisfactory composite reliability of the latent constructs (Hair et al. 2019). As no external criterion instrument was administered alongside the CRCDCS, this convergent validity evidence is model‐based (AVE‐derived) rather than criterion‐related; assessment of convergent validity against an independent, previously validated instrument remains an important direction for future research. Discriminant validity, evaluated through inter‐factor correlations relative to the square root of each factor's AVE (Fornell and Larcker 1981), indicated that each factor measured a conceptually distinct aspect of critical reflective competence, despite the substantive theoretical relationships among the three dimensions. Full factor correlation statistics supporting this assessment are reported in Table 4. Such differentiation is particularly relevant for multidimensional constructs associated with clinical judgement and professional decision‐making (Asselin and Fain 2013; Levett‐Jones et al. 2010).

The CRCDCS demonstrated satisfactory internal consistency, as indicated by Cronbach's alpha and McDonald's omega coefficients across all three dimensions (Dunn et al. 2014; Hayes and Coutts 2020). The AECR dimension demonstrated the highest reliability, which may reflect the more structured and cognitively oriented nature of analytical processes in delirium care (Fick et al. 2002; Oh et al. 2017), whereas the ethical and preventive dimensions, being more susceptible to contextual and experiential influences, showed somewhat greater response variability. The satisfactory ICC value further demonstrated acceptable temporal stability of the instrument (DeVon et al. 2007; Koo and Li 2016).

As a complementary approach to internal structure, EGA confirmed the three‐factor structure and demonstrated that the construct dimensions function as an interactive, dynamic network, consistent with the complex and multilayered nature of clinical reflection, in which analytical reasoning, ethical reflection, and predictive thinking mutually influence one another (Asselin and Fain 2013; Epskamp et al. 2018; Golino and Epskamp 2017; Mann et al. 2009). Satisfactory TEFI values supported the adequacy of the estimated network structure (Christensen and Golino 2021; Golino et al. 2022).

As a supplementary exploratory analysis, random forest modeling was used to examine the relative contribution of individual items and first‐order dimensions to the internal score structure of the CRCDCS. Random forest models are primarily predictive algorithms, and their variable‐importance estimates should be interpreted in relation to the outcome and predictor specification rather than as direct evidence of construct or criterion‐related validity (Biau and Scornet 2016; Breiman 2001). In the present study, both the predictors and the outcome were derived from the same set of CRCDCS items; therefore, the analysis reflects the internal item‐to‐total‐score structure of the instrument rather than external predictive validity. Findings indicated that several items related to clinical analysis and reconsideration of care decisions showed relatively greater contributions to the internal score structure of the CRCDCS. At the dimensional level, AECR showed the highest relative contribution among the three subscales. These findings should therefore be interpreted as descriptive information regarding the internal structure of the instrument rather than as evidence that the CRCDCS predicts independent clinical or professional outcomes.

Taken together, the present findings provide substantial evidence supporting the internal structure and reliability of the CRCDCS, together with model‐based evidence relevant to convergent and discriminant validity. Evidence for predictive validity was not established in the present study, as the ability of the CRCDCS to predict independent clinical or professional outcomes was not directly examined. Importantly, evidence based on internal model characteristics should not be equated with criterion‐related or predictive validity, which requires an appropriate external criterion or outcome (American Educational Research Association 2014; DeVellis and Thorpe 2021). Accordingly, external and predictive validity should be examined in future studies using independent clinical or professional outcomes.

## 5 Conclusion

The findings of the present study provide substantial psychometric evidence supporting the multidimensional internal structure and reliability of the CRCDCS for assessing critical reflective competence among nurses working in intensive care settings. EFA, parallel analysis, CFA, and EGA converged in supporting a three‐factor structure comprising AECR, PCER, and ROPR. Random forest analysis provided supplementary descriptive information regarding the relative contribution of individual items and dimensions to the internal score structure of the instrument. Together, these findings support the structural coherence and reproducibility of the three‐dimensional configuration identified in the present sample.

Evidence from AVE, composite reliability, inter‐factor correlations, and complementary reliability indices further supported the internal psychometric properties of the instrument. However, these findings should be interpreted within the scope of the evidence generated by the present study. In particular, the study did not directly assess predictive validity or criterion‐related validity against independent clinical or professional outcomes, and the model‐based evidence for convergent validity should not be equated with external criterion validity. Accordingly, the CRCDCS should be considered a promising instrument with evidence supporting its internal structure and reliability, while further longitudinal and external‐validation studies are needed to establish its predictive, criterion‐related, and broader construct validity.

The three dimensions identified in the CRCDCS capture analytically oriented reflection, person‐centered and ethical reflection, and risk‐oriented preventive reasoning, respectively. Although AECR accounted for the largest proportion of explained variance and showed a relatively greater contribution to the internal score structure, these findings do not establish that AECR is theoretically or clinically more important than PCER or ROPR. Rather, they indicate that analytical reflection was relatively prominent within the empirical structure observed in this sample. The three dimensions should therefore be interpreted as complementary components of critical reflective competence in delirium care, with their relative clinical significance requiring further investigation using external clinical, professional, and longitudinal outcomes.

## 6 Strengths and Limitations

To the best of our knowledge, this study is the first to design and validate the CRCDCS with the aim of multidimensionally assessing nurses' critical reflective competence in the management of patients with delirium. One of the major strengths of this study was the construct‐driven development of the instrument through the integration of both theoretical and empirical evidence. Specifically, the initial items were generated through a systematic review of the relevant scientific literature and qualitative content analysis of semi‐structured interviews conducted with nurses working in intensive care units. This approach ensured that the conceptual dimensions of the instrument were grounded not only in theoretical foundations but also in the clinical realities and professional experiences of nurses. Another important strength of the study was the simultaneous use of advanced and complementary psychometric approaches. The application of EFA, CFA, and EGA, together with comprehensive reliability assessment through *α*, *ω*, ICC, and the standard error of measurement, strengthened the evaluation of the validity, stability, and precision of the instrument, while random forest modeling provided supplementary exploratory information regarding its internal score structure. Moreover, the incorporation of network‐based approaches and machine learning techniques distinguishes the present study from many conventional instrument‐development studies and provides deeper insights into the structure and functioning of the construct.

Despite these strengths, the present study also has several limitations. First, the cross‐sectional design limited the ability to examine longitudinal changes in critical reflective competence and to evaluate the sensitivity of the instrument to educational interventions over time. Second, the study sample consisted exclusively of nurses working in intensive care units of teaching hospitals affiliated with two universities of medical sciences in Iran. Therefore, the generalisability of the findings to other healthcare settings, cultures, and healthcare systems requires further investigation. Third, the data were collected using a self‐report instrument, which may have been influenced by response bias and social desirability bias, thereby partially limiting the accurate estimation of actual professional performance. Accordingly, future studies are recommended to employ longitudinal designs, multicenter samples, and a combination of observational data and objective indicators of clinical performance to further evaluate the psychometric properties and clinical applicability of the CRCDCS. Fourth, external criterion‐related validity of the CRCDCS was not assessed, as no comparable instrument was administered concurrently; future studies should examine the correlation of the CRCDCS with related, previously validated instruments (e.g., Shin et al.'s Critical Reflection Competency Scale) to further support its convergent validity.

Additionally, the three‐level competency categorisation was based on an equal‐range division of the total score rather than empirically derived or clinically validated cut‐off points; future research using distribution‐based methods (e.g., percentile ranking) or criterion‐based approaches (e.g., ROC curve analysis against an external clinical indicator) is recommended to establish evidence‐based thresholds. Consequently, although AECR accounted for the largest proportion of explained variance in the exploratory factor structure, the present findings do not establish its greater theoretical or clinical importance relative to PCER and ROPR. Future studies should examine the associations of the individual CRCDCS dimensions with relevant clinical, professional, and patient‐related outcomes to determine their incremental and criterion‐related validity. Fifth, measurement invariance of the CRCDCS across relevant participant characteristics was not examined in the present study. Therefore, it remains unclear whether the instrument functions equivalently across different subgroups, and future research is recommended to assess configural, metric, and scalar invariance across key demographic and professional variables.

## 7 Relevance to Clinical Practice

The CRCDCS is a multidimensional instrument developed to assess nurses' critical reflective competence in the care of patients with delirium, demonstrating sound internal structure, internal consistency, and test–retest reliability in this sample. Pending further establishment of external criterion validity, predictive validity, and measurement invariance, the instrument may hold potential for supporting the identification of performance gaps and evaluation of nurses' professional preparedness in delirium management. It may also offer a starting point for evaluating educational programs and specialised training aimed at strengthening reflective thinking in critical care settings, although its responsiveness to such interventions has not yet been tested. The differentiation of analytical, ethical, and anticipatory dimensions of professional competence may help nursing managers and healthcare policymakers consider educational and organisational priorities in a more structured way. Continued psychometric evaluation across independent and longitudinal samples is recommended before the CRCDCS is used for monitoring care quality or evaluating the effectiveness of educational interventions in practice.

## Author Contributions

**Fahimeh Magsoodi:** conceptualization, investigation, validation, writing – original draft, data curation. **Fatemeh Ghaffari:** conceptualization, writing – original draft, writing – review and editing, validation, methodology, software, formal analysis, supervision, project administration.

## Funding

This study was funded by Babol University of Medical Sciences. The funder had no role in study design, data collection, data analysis, or the decision to publish or prepare the manuscript.

## Ethics Statement

Informed voluntary consent was obtained from all participants in both the qualitative and quantitative phases of the study. The study was conducted in accordance with the Declaration of Helsinki and was approved by the Ethics Committee of Babol University of Medical Sciences (Code: IR.MUBABOL.REC.1404.030; approval date: May 12, 2025) as part of a broader institutional research project (Contract No. 724136621). This approval covered both the qualitative and quantitative phases of the study.

## Consent

The authors have nothing to report.

## Conflicts of Interest

The authors declare no conflicts of interest.

## Supporting information


**Table S1:** Pattern matrix of the exploratory factor analysis (EFA).

**Appendix A.** Final 19‐item version of the Critical Reflection Competency in Delirium Care Scale (CRCDCS).

## Data Availability

The original contributions presented in the study are included in the article/Supporting Information, and further inquiries can be directed to the corresponding author.
