# Supplementary material for: Development and Psychometric Evaluation of the Critical Reflection Competency in Delirium Care Scale (CRCDCS) Among Iranian ICU Nurses
Source: Nurs Open. 2026 Sep 18;13(9):e70818. doi: 10.1002/nop2.70818 (PMC13588630; doi:10.1002/nop2.70818)
Supplement: Supplementary file 1 [file NOP2-13-e70818-s001.docx]

Appendix A. Final 19-item version of the Critical Reflection Competency in Delirium Care Scale (CRCDCS).

| No. | Item | 1 | 2 | 3 | 4 | 5 |
| --- | --- | --- | --- | --- | --- | --- |
|  |  | Never | Rarely | Sometimes | Often | Always |
| Analytical–Evaluative Clinical Reflection (AECR) | | | | | | |
| 1 | Following the assessment of patients with delirium based on specific protocols, I analyse the causes of deviations from these protocols within the healthcare team and document the findings. | □ | □ | □ | □ | □ |
| 2 | By reviewing delirium-related care events, I separately analyse the factors influencing sustained cognitive improvement or deterioration, as well as acute clinical factors, and document the findings. | □ | □ | □ | □ | □ |
| 3 | Through systematic evaluation of medication management, environmental conditions, and interprofessional interactions, I identify practices inconsistent with established evidence and document specific corrective recommendations to improve team performance. | □ | □ | □ | □ | □ |
| 4 | By comparing the trajectory of delirium severity and disease progression with the patient's individual characteristics, I identify and document key factors requiring reconsideration of clinical interventions. | □ | □ | □ | □ | □ |
| 5 | When screening results are inconsistent with the patient's clinical presentation, I analyse the possible causes of this discrepancy and document the findings to improve the accuracy of future assessments. | □ | □ | □ | □ | □ |
| 6 | By analysing cognitive and functional documentation of patients with delirium after discharge, I identify areas requiring revision in care protocols and formally document corrective recommendations. | □ | □ | □ | □ | □ |
| 7 | By comparing patients' cognitive, functional, and survival outcomes with initial predictions, I identify the causes of discrepancies and use the findings to refine future clinical decision-making. | □ | □ | □ | □ | □ |
| Person-Centered Ethical Reflection (PCER) | | | | | | |
| 8 | During the review of care practices, I evaluate the extent to which respect, privacy, and physical comfort of patients with delirium were maintained based on observations and documented data, and I identify areas of deficiency. | □ | □ | □ | □ | □ |
| 9 | Following each interaction with the family of a patient with delirium, I compare changes in their level of concern or anxiety based on observations or reports and identify the reasons in order to improve future communication strategies. | □ | □ | □ | □ | □ |
| 10 | After implementing an intervention, I reflect on its ethical and humanistic consequences in light of disease severity, level of consciousness, and care limitations, and document the findings to improve ethical approaches in similar future situations. | □ | □ | □ | □ | □ |
| 11 | I analyse patient records and family reports to identify sociocultural patterns influencing interaction and cooperation, and I apply the findings in designing communication and supportive interventions. | □ | □ | □ | □ | □ |
| 12 | I analyse the influence of patients' or families' stated preferences and perspectives on care decisions and use the findings to improve the healthcare team's decision-making process in similar future situations. | □ | □ | □ | □ | □ |
| 13 | After implementing care interventions, I reflect on their impact on the comfort, dignity, and sense of safety of patients with delirium from the perspective of their lived experiences and family reports, and I use the findings to improve person-centered care approaches. | □ | □ | □ | □ | □ |
| Risk-Oriented Preventive Reflection (ROPR) | | | | | | |
| 14 | Upon admission of high-risk patients, I identify, prioritise, and document predisposing factors for delirium. | □ | □ | □ | □ | □ |
| 15 | I identify early behavioural or cognitive signs, such as transient attention deficits, and predict the onset of delirium by documenting and monitoring changes over time. | □ | □ | □ | □ | □ |
| 16 | By reviewing the patient's care plan, I identify interventions that may contribute to the development or exacerbation of delirium and document the rationale for selecting or excluding each intervention. | □ | □ | □ | □ | □ |
| 17 | By comparing documented data from similar patients, I identify predictive patterns of delirium and apply them in designing preventive interventions for future patients. | □ | □ | □ | □ | □ |
| 18 | I analyse sequential physiological and cognitive changes in patients prior to the onset of delirium and document early warning indicators as future monitoring patterns. | □ | □ | □ | □ | □ |
| 19 | I compare and analyse the results of repeated screenings in high-risk patients to identify patterns of risk changes and, accordingly, revise and refine future monitoring and preventive intervention plans. | □ | □ | □ | □ | □ |
