# Supplementary material for: Development and Psychometric Evaluation of the Critical Reflection Competency in Delirium Care Scale (CRCDCS) Among Iranian ICU Nurses
Source: Nurs Open. 2026 Sep 18;13(9):e70818. doi: 10.1002/nop2.70818 (PMC13588630; doi:10.1002/nop2.70818)
Supplement: Supplementary file 2 [file NOP2-13-e70818-s002.docx]

**Supplementary Table S1. Pattern Matrix of the Exploratory Factor Analysis (EFA)**

| **Item** | **Factor 1** | **Factor 2** | **Factor 3** |
| --- | --- | --- | --- |
| Item 1 | .683 | .002 | -.025 |
| Item 2 | .746 | .026 | -.007 |
| Item 3 | .719 | .006 | .012 |
| Item 4 | .853 | -.094 | .023 |
| Item 5 | .845 | -.012 | .016 |
| Item 6 | .633 | .039 | -.008 |
| Item 7 | .700 | .065 | -.016 |
| Item 8 | -.036 | .802 | .009 |
| Item 9 | .067 | .732 | .016 |
| Item 10 | -.013 | .733 | .061 |
| Item 11 | .035 | .838 | -.058 |
| Item 12 | .013 | .757 | .000 |
| Item 13 | -.036 | .830 | -.014 |
| Item 14 | -.066 | .070 | .758 |
| Item 15 | -.029 | .021 | .718 |
| Item 16 | -.001 | -.067 | .826 |
| Item 17 | .025 | -.100 | .750 |
| Item 18 | .038 | .050 | .685 |
| Item 19 | .036 | .055 | .733 |

*Note:* Extraction Method: Principal Axis Factoring. Rotation Method: Promax with Kaiser Normalization. a. Rotation converged in 5 iterations.
